# The effect of cashew leaf extract on small intestine morphology and growth performance of *Jawa Super* chicken

**DOI:** 10.14202/vetworld.2018.1047-1054

**Published:** 2018-08-03

**Authors:** H. Setiawan, M. E. Jingga, H. T. Saragih

**Affiliations:** 1 Laboratory of Biology, Faculty of Mathematics and Science, Universitas Ahmad Dahlan, Yogyakarta, 55166, Indonesia; 2 Laboratory of Animal Development Structure, Faculty of Biology, Universitas Gadjah Mada, Yogyakarta, 55281, Indonesia

**Keywords:** cashew leaf, feed supplement, growth performance, *Jawa Super* chicken, small intestine

## Abstract

**Aim:**

This research aimed to study the effect of leaf extract of cashew as a bioactive compound in feed on the morphology of the small intestine in *Jawa Super* chicken (*Gallus gallus domesticus*).

**Materials and Methods:**

This study used 72 1-day-old *Jawa Super* chicks reared for a further 16 days. We used a randomized complete design, in which basal feed was supplemented with ethanolic extract of cashew leaves at 0 g/kg feed (control), 1.25 g/kg feed (P1), 2.5 g/kg feed (P2), 5 g/kg feed (P3), 10 g/kg feed (P4), and 20 g/kg feed (P5). Parameters observed included growth performance, chicken morphometry, and morphology of the small intestine, comprising the length and width of the villi, the depth of the crypt, and the number and size of goblet cells in the duodenum, jejunum, and ileum. Data analysis was conducted using one-way ANOVA followed by Duncan’s test, with significance defined as p<0.05.

**Results:**

Ethanolic extract of cashew leaf significantly increased body weight, feed efficiency, body morphometry, villus length, crypt depth, number of goblet cells, and extent of goblet cell area of the small intestine at 16 days. The morphological results from the small intestine showed that P4 and P5 were significantly better than control.

**Conclusions:**

Cashew leaf ethanolic extract mixed with 10 g/kg basal feed is effective as a natural feed supplement for *Jawa Super* chickens.

## Introduction

Antibiotics are used widely in the livestock industry to reduce the growth of pathogens and the spread of diseases and to improve the quality of meat and eggs [1,2]. The European Union has banned the use of synthetic antibiotics in livestock through either injection, drinking water, or mixed feed because it results in bacterial resistance and leaves residues in meat/chicken products [3].

In Indonesia, demand for chicken meat increases every year. One type of chicken that is in high demand by the livestock industry in Indonesia is the *Jawa Super* chicken. The *Jawa Super* chicken is a cross between a broiler laying chicken and a male *Kampoeng* chicken and thus has the nature of a *Kampoeng* chicken and a broiler chicken. *Jawa Super* chicken meat also has a distinctive taste similar to the *Kampoeng* chicken and low-fat content [4]. However, the productivity of *Jawa Super* chicken cannot be maximized if the animals are not provided with high-quality feed. Good feed quality is supported by a feed supplement that serves as an antimicrobial growth promoter. The animal feed industry in Indonesia needs alternatives to synthetic antibiotics for the use as animal feed additives.

Natural antimicrobials can be found in various plants, including cashew (*Anacardium occidentale* L.). Ethanolic extracts of cashew leaf contain secondary metabolites such as flavonoids, tannins, saponins, anthocyanins, and alkaloids. In *in vitro* experiments, tannin in cashew leaf can act as an antimicrobial and fungicidal substance [5]. Compounds such as flavonoids and quercetin in cashew leaves are also known as natural antimicrobials that can protect the body from pathogen attack [6].

One way to determine whether antimicrobial compounds have a good influence on livestock growth is by examining the morphology of the small intestine. Antimicrobials can increase the thickness of the gastrointestinal tract and increase the number of mucous glands, which improve nutrient absorption of feed [1]. Antimicrobials in natural feed supplements can increase the area of the villi and the depth of the crypts in the small intestine and increase nutrient absorption from the feed [7]. The content of compounds such as flavonoids, quercetin, and tannins in cashew leaf ethanolic extracts may, therefore, influence the growth and morphology of the *Jawa Super* chicken’s small intestine.

Research on the influence of cashew ethanolic extract on chicken feed has not been conducted, so this study aimed to determine the potency of cashew leaf extract (CLE), as a supplement in chicken feed that functions as a natural antimicrobial agent.

## Materials and Methods

### Ethical approval

This research used a standard procedure that has been certified by the ethical board of the Integrated Research and Testing Laboratory of Gadjah Mada University with certification number 00005/04/LPPT/II/2017.

### Ethanol extract of cashew leaf

Fresh cashew leaves were sliced into small pieces and dried in an oven at 50°C for 24 h. They were then smoothed/milled with a blender to facilitate the extraction process. The powder was weighed and then macerated with 96% ethanol. The liquid extract was then evaporated until it was free of ethanol solution using a vacuum evaporator (Rotary evaporator) at 60°C for 3 h until the extract was viscous.

### Birds and formulation of basal feed

Basal feed (Table-1) was chicken starter feed with ~21% protein content. This was mixed with cashew ethanolic extract. The following concentrations were analyzed: Control (0 g of CLE/kg of basal feed), P1 (1.25 g of CLE/kg of basal feed), P2 (2.5 g of CLE/kg of basal feed), P3 (5 g of CLE/kg of basal feed), P4 (10 g of CLE/kg of basal feed), and P5 (20 g of CLE/kg of basal feed).

**Table-1:** Composition of basal feed.

Composition of feed (%)	Starter					

	CLE (g/kg)					

	0	1.25	2.5	5	10	20
Corn	63.53	63.53	63.53	63.53	63.53	63.53
Soybean meal	28.38	28.38	28.38	28.38	28.38	28.38
Meat and bone meal	4.70	4.70	4.70	4.70	4.70	4.70
Crude palm oil	0.95	0.95	0.95	0.95	0.95	0.95
Dicalcium phosphate	0.67	0.67	0.67	0.67	0.67	0.67
Premix mensa	0.50	0.50	0.50	0.50	0.50	0.50
Steamed bone meal	0.45	0.45	0.45	0.45	0.45	0.45
D, L-methionine	0.23	0.23	0.23	0.23	0.23	0.23
NaCl	0.22	0.22	0.22	0.22	0.22	0.22
CaCO_3_	0.18	0.18	0.18	0.18	0.18	0.18
L-lysine HCl	0.10	0.10	0.10	0.10	0.10	0.10
L-threonine	0.08	0.08	0.08	0.08	0.08	0.08
Toxin binder	0.01	0.01	0.01	0.01	0.01	0.01
Composition calculation (unit)	Total	Total	Total	Total	Total	Total
Weight (kg)	1.00	1.00	1.00	1.00	1.00	1.00
Dry matter (%)	87.04	87.04	87.04	87.04	87.04	87.04
Moisture (%)	13.0	13.0	13.0	13.0	13.0	13.0
Metabolism energy poultry (kcal/kg)	3,000.343	3,000.343	3,000.343	3,000.343	3,000.343	3,000.343
Crude protein (%)	21.000	21.000	21.000	21.000	21.000	21.000
Crude fat (%)	4.34	4.34	4.34	4.34	4.34	4.34
Fiber (%)	1.57	1.57	1.57	1.57	1.57	1.57
Lysine (%)	1.212	1.212	1.212	1.212	1.212	1.212
Methionine	0.667	0.667	0.667	0.667	0.667	0.667
Methionine+cysteine (%)	1.032	1.032	1.032	1.032	1.032	1.032
Threonine (%)	0.935	0.935	0.935	0.935	0.935	0.935
Tryptophan (%)	0.251	0.251	0.251	0.251	0.251	0.251
Arginine (%)	1.371	1.371	1.371	1.371	1.371	1.371
Isoleucine (%)	0.876	0.876	0.876	0.876	0.876	0.876
Valine (%)	1.034	1.034	1.034	1.034	1.034	1.034
Calcium (%)	0.900	0.900	0.900	0.900	0.900	0.900
Phosphorus, total (%)	0.749	0.749	0.749	0.749	0.749	0.749
Phosphorus, available (%)	0.400	0.400	0.400	0.400	0.400	0.400
Sodium (%)	0.134	0.134	0.134	0.134	0.134	0.134
Chloride (%)	0.209	0.209	0.209	0.209	0.209	0.209

CLE=Cashew leaf extract

### Chicken feed acclimatization, maintenance, and feeding

In this research, 72 1-day-old chicks of *Jawa Super* chickens were divided into six groups, each group consisting of 12 chickens. Acclimatization was conducted for 3 days from day 0 to day 2, and each group was maintained in a 60 cm×50 cm×50 cm box equipped with an incandescent lamp to keep the temperature warm. Treatment began at the age of the day 3 and continued to day 16. Chickens were given feed and drinking water *ad libitum*. Temperature was measured daily. Cages were cleaned every 3 days. The chicks were weighed at the age of 0, 3, 6, 9, 12, 15, and 16 days.

### Calculation of feed conversion ratio (FCR)

The amount of feed given was calculated daily until the age of day 16. Feed intake (g/day) was obtained from the total weight of feed given minus the total weight of the residual feed. The feed intake (g/day) and weight gain (g/day) were then divided by the number of chickens. The formula for calculating FCR is as follows:


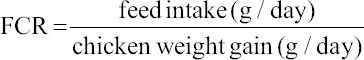


### Euthanasia and organ preparation

When chickens reached day 16, they were fasted for 12 h. Subsequently, five chickens per group were sacrificed by dislocation of the neck. Chickens were dissected in the ventral section using scissors and a scalpel. The small intestine was separated from other organs, washed with physiological saline (0.9% NaCl), and weighed. The length of the intestine was measured, and the duodenum, ileum, and jejunum of the small intestine were then separated. Each organ was inserted into Bouin’s solution for histological preparation [8,9].

### Histological preparation of the small intestine

The small intestine was histologically prepared using Bouin’s, alcohol, toluol, paraffin, xylol, hematoxylin-eosin (HE) dye solutions, periodic acid–Schiff (PAS)–Alcian blue (AB), and Image Raster software version 4.0.5. The paraffin method was utilized. The duodenum, jejunum, and ileum were then fixed using Bouin’s solution for 12 h. In this research, two kinds of stains, namely HE and PAS–AB, were used; HE staining was used to measure the villi and depth of the crypt, whereas PAS–AB staining was used to calculate the number and extent of goblet cells in the small intestine [10,11].

### Morphology of the small intestine

Observations were made using light microscopy (100× and 400× magnification). Images of the intestine were obtained using the camera device of an AmScope microscope. Observations of the intestine included the following steps:

#### Measurement of the length, width, and area of the villi

The length, basal width, and apical width of the villi in the duodenum, jejunum, and ileum were calculated using a microscope at 100× magnification. Image capture used AmScope with five viewing fields for each preparation. The length and width of villi were measured using a computer with the Image Raster program. The area of villi (µm^2^) was calculated using the following formula [12]:


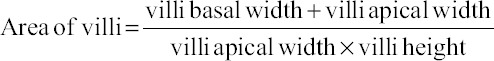


#### Measurement of crypt depth (CD)

The depth of the crypt was observed in five different fields of view in one histological slide. The comparative ratio between the length of the villi and the depth of the crypts was calculated by the following formula [12]:


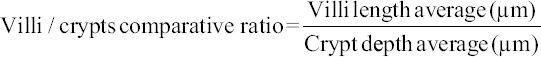


### Calculation of the number and area of goblet cells

The number of goblet cells was calculated in the duodenum, jejunum, and ileum villi using 400× magnification. The number of goblet cells was determined by counting goblet cells per 100 μm villus length (VL) [1]. Calculations were done on five fields of view on each villus. The area of the goblet cells was determined by measuring from the edges of the membrane surrounding the goblet cell “cup” on the cross section of the small intestine villi [13].

### Statistical analysis

Weight data, FCR, and small intestine morphology were analyzed using one-way ANOVA, followed by least significant difference and Duncan’s test at the 5% confidence level.

## Results

### Growth of Jawa Super chickens

Our data revealed that the weight of *Jawa Super* chickens treated with CLE exhibited significant differences between the control and treatment groups at days 6-16, except the P2 group (p<0.05) (Table-2). FCR in *Jawa Super* chickens administered feed-containing CLE improved in the P4 and P5 groups compared with that in the control, P1, P2, and P3 groups. Thus, CLE can positively influence weight gain and feed efficiency in *Jawa Super* chickens until at least 16 days of age.

**Table-2:** Growth data for each CLE treatment group on basal feed in *Jawa Super* chickens from day 0 to day 16 of age.

Variable	Age	Treatment					

		Control	P1	P2	P3	P4	P5
Body weight (g)	Day 0	36.17±2.82^ns^	36.25±1.86^ns^	36.50±1.51^ns^	36.67±2.77^ns^	36.50±1.83^ns^	36.25±1.36^ns^
	Day 3	42.50±5.63^ns^	41.00±4.59^ns^	40.75±3.05^ns^	40.92±3.58^ns^	41.08±3.53^ns^	41.58±1.38^ns^
	Day 6	51.83±4.17^a^	54.00±6.13^b^	45.58±5.74^b^	52.75±4.18^b^	55.08±4.83^b^	53.08±6.47^b^
	Day 9	56.25±10.10^ab^	60.66±6.54b^c^	51.58±7.77^a^	61.08±5.19^bc^	66.67±5.61^c^	67.00±9.37^c^
	Day 12	77.00±16.06^b^	88.33±12.93^c^	65.33±13.48^a^	88.75±16.03^c^	88.58±6.70^c^	97.33±10.27^c^
	Day 15	90.83±16.80^ab^	94.67±13.54^b^	81.25±18.45^a^	110.92±9.28^c^	113.50±8.32^c^	115.75±13.36^c^
	Day 16	94.75±18.93^ab^	102.16±15.84^b^	88.25±18.90^a^	124.08±11.46^c^	125.08±9.31^c^	123.00±12.9^c^
Feed intake (g/day)		14.65±0.89^cd^	12.41±0.486^ab^	13.07±1.46^abc^	14.06±1.57^bcd^	15.21±1.91^d^	11.89±1.28^a^
Weight gain (g/day)		9.90±4.17^ns^	10.71±4.21^ns^	8.92±3.58^ns^	11.28±3.53^ns^	11.67±3.80^ns^	11.75±3.40^ns^
FCR		1.62±0.46^b^	1.26±0.37^a^	1.57±0.36^b^	1.30±0.21^a^	1.35±0.24^a^	1.08±0.36^a^

Control: Group with basal feed; P1: Basal feed+CLE, 1.25 g/kg of feed; P2: Basal feed+CLE, 2.5 g/kg of feed; P3: Basal feed+CLE, 5 g/kg of feed; P4: Basal feed+CLE, 10 g/kg of feed; P5: Basal feed+CLE, 20 g/kg of feed. Mean±SD, ^a-c^ddifferent letters on values in the same row indicate a significant difference (p<0.05), ^ns^values do not differ significantly (p≥0.05). CLE=Cashew leaf extract, SD=Standard deviation, FCR=Feed conversion ratio

### Duodenum morphology

**Figure-1:**
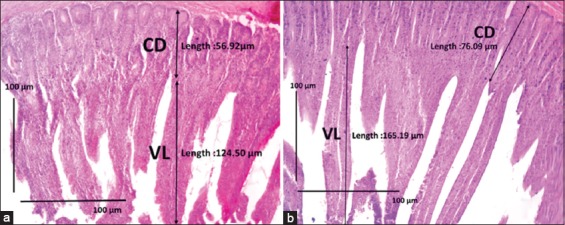
Sections of duodenum tissue of *Jawa Super* chicken. (a) Control group with basal feed. (b) Chicks treated with basal feed + cashew leaf extract (CLE) (10 g/kg of feed; P4 group). In the control, the villi are shorter and thicker than those in chicks fed CLE. CD=Crypt depth, VL=Villus length. Hematoxylin-eosin staining. Scale bars represent 100 µm.

**Figure-2:**
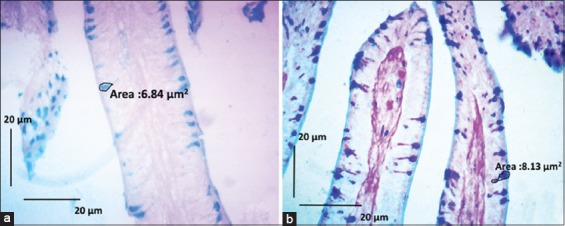
Sections of duodenum tissue of *Jawa Super* chicken. (a) Control group with basal feed. (b) Chicks treated with basal feed + cashew leaf extract (CLE) (10 g/kg of feed; P4 group). In the control, the goblet cells are smaller than those in chicks fed CLE. CD=Crypt depth, VL=Villus length. Periodic acid–Schiff–Alcian blue staining. Scale bars represent 20 µm.

The analysis of the duodenum morphology of *Jawa Super* chickens at day 16 treated with CLE (Table-3) showed that the P4 villi were significantly longer than the others (Figure-1). Meanwhile, the crypts in the duodenum were also significantly deeper in P3 than in the control group. The area of the duodenal villi in P4 and P5 was significantly larger than that in the control. The ratio of villi/crypts of the duodenum in the P4 and P5 groups was significantly larger compared with the control. The number of goblet cells in the duodenum in P4 was significantly higher than that in the control, and the area of these goblet cells was also significantly larger in P4 than in the control (Figure-2).

**Table-3:** Data for VL, CD, villi/crypts ratio, area of villi, and number and area of duodenum goblet cells in each CLE treatment group on the *Jawa Super* chicken basal feed at day 16.

Variable	Treatment					

	Control	P1	P2	P3	P4	P5
Villus length (μm)	140.38±9.66^a^	148.84±14.83^a^	148.76±20.92^a^	146.19±11.44^a^	218.55±66.06^b^	215.43±40.94^b^
Crypt depth (μm)	87.36±13.06^ab^	76.55±14.18^a^	80.12±9.63^ab^	96.24±20.01^b^	87.69±6.81^ab^	73.08±5.20^a^
Villi/crypts ratio	1.66±0.25^a^	2.20±0.51^ab^	2.30±0.63^ab^	1.92±0.58^a^	2.78±0.83^b^	2.83±0.15^b^
Area of villus (μm^2^)	287.31±20.39^a^	335.72±46.61^a^	356.63±70.29^a^	350.92±46.27^a^	634.38±204.87^b^	536.86±119.35^b^
Number of goblet cells	83.00±3.72^a^	91.12±4.80^ab^	91.52±14.86^ab^	93.84±8.33^ab^	102.64±8.90^b^	89.60±13.91^ab^
Area of goblet cell (μm^2^)	5.66±1.04^a^	5.89±0.92^a^	6.86±2.96^a^	7.44±0.32^ab^	11.40±1.76^c^	9.38±1.88^bc^

Control: Group with basal feed; P1: Basal feed+CLE, 1.25 g/kg of feed; P2: Basal feed+CLE, 2.5 g/kg of feed; P3: Basal feed+CLE, 5 g/kg of feed; P4: Basal feed+CLE, 10 g/kg of feed; P5: Basal feed+CLE, 20 g/kg of feed. Mean±SD, ^a-c^different letters on values in the same row indicate a significant difference (p<0.05), ^ns^values do not differ significantly (p≥0.05). CLE=Cashew leaf extract, SD=Standard deviation, VL=Villus length, CD=Crypt depth

### Jejunum morphology

Our investigation of jejunum morphology of *Jawa Super* chickens at day 16 treated with CLE (Table-4) showed that the P4 villi were significantly longer than the others. On the other hand, the CD in the jejunum was not significantly different, for any CLE-treated group, from that of the control (p<0.05). The area of the villi also did not differ significantly between treatments, although the ratio of villi/crypts of the jejunum in the P4 and P5 groups was significantly larger compared with the control. The number of goblet cells in the jejunum in P3 and P4 was significantly higher than that in the control. The area of goblet cells in the jejunum was also significantly larger in P4 and P5 than in the control.

**Table-4:** Data for VL, CD, villi/crypts ratio, area of villi, and number and area of jejunum goblet cells in each CLE treatment group on *Jawa Super* chicken basal feed at 16 days of age.

Variable	Treatment					

	Control	P1	P2	P3	P4	P5
Villus length (μm)	142.02±12.69^a^	176.03±25.80^ab^	177.78±46.82^ab^	179.51±16.02^ab^	186.45±40.12^b^	173.28±11.35^ab^
CD (μm)	67.74±17.95^ns^	69.17±17.35^ns^	82.21±12.43^ns^	84.59±20.33^ns^	78.06±17.14^ns^	86.52±7.37^ns^
Villi/crypts ratio	2.26±0.54^ns^	2.44±0.62^ns^	2.51±0.57^ns^	2.52±0.82^ns^	2.81±0.28^ns^	2.07±0.27^ns^
Area of villus (μm^2^)	329.11±47.21^ns^	435.61±99.35^ns^	384.34±99.59^ns^	380.01±47.74^ns^	415.77±86.16^ns^	412.69±50.43^ns^
Number of goblet cells	72.88±11.20^a^	74.24±7.35^ab^	80.80±6.31^ab^	100.76±9.15^c^	111.20±12.59^c^	86.56±8.29^b^
Area of goblet cell (μm^2^)	5.95±0.73^a^	6.48±1.50^a^	6.35±1.66^a^	6.72±1.29^a^	9.51±1.16^b^	9.32±0.69^b^

Control: Group with basal feed; P1: Basal feed+CLE, 1.25 g/kg of feed; P2: Basal feed+CLE, 2.5 g/kg of feed; P3: Basal feed+CLE, 5 g/kg of feed; P4: Basal feed+CLE, 10 g/kg of feed; P5: Basal feed+CLE, 20 g/kg of feed. Mean±SD, ^a^cdifferent letters on values in the same row indicate a significant difference (p<0.05), ^ns^values do not differ significantly (p≥0.05). CLE=Cashew leaf extract, SD=Standard deviation, VL=Villus length, CD=Crypt depth

### Ileum morphology

Ileum morphology of *Jawa Super* chickens at day 16 treated with CLE (Table-5) was not significantly different for VL between the control and any of the treatment groups. Meanwhile, the crypts in the ileum were significantly deeper in P2 and P3 than in the control group. The area of the duodenal villi in P4 and P5 was significantly larger than that in the control, and the ratio of villi/crypts of the ileum in these two groups was significantly larger than in the control. Villus area in the ileum was also significantly larger in P2 and P5 than in the control. The number of goblet cells in the ileum in P4 was significantly higher than that in the control, while the area of goblet cells in the ileum was also significantly larger in P4 than in the control.

**Table-5:** Data for VL, CD, villi/crypts ratio, area of villi, and number and area of goblet cells of the ileum in each CLE treatment group on *Jawa Super* chicken basal feed at 16 days of age

Variable	Treatment					

	Control	P1	P2	P3	P4	P5
Villus length (μm)	133.38±12.07^ns^	148.15±14.77^ns^	151.05±24.01^ns^	157.01±20.56 *^ns^*	163.08±17.01^ns^	149.24±31.88^ns^
CD (μm)	69.38±15.76^a^	78.65±6.96^ab^	83.79±6.42^ab^	85.62±9.74 *^ab^*	89.98±15.24^b^	91.91±17.57^b^
Villi/crypts ratio	1.88±0.40^ns^	1.9±0.22^ns^	1.90±0.32^ns^	2.16±0.23 *^ns^*	2.24±0.53^ns^	1.91±0.60^ns^
Area of villus (μm^2^)	290.39±22.61^a^	379.96±63.11^b^	350.24±70.18^ab^	392.66±53.72*^b^*	370.02±15.21^b^	363.46±79.48^ab^
Number of goblet cells	90.04±7.13^a^	96.08±7.08^ab^	90.56±8.94^a^	96.16±13.13 *^ab^*	131.96±7.88^c^	102.92±4.66^b^
Area of goblet cell (μm^2^)	6.40±1.36^a^	6.31±1.55^a^	8.19±0.82^b^	8.32±1.37 *^b^*	15.14±1.63^d^	12.70±0.80^c^

Control: Group with basal feed; P1: Basal feed+CLE, 1.25 g/kg of feed; P2: Basal feed+CLE, 2.5 g/kg of feed; P3: Basal feed+CLE, 5 g/kg of feed; P4: Basal feed+CLE, 10 g/kg of feed; P5: Basal feed+CLE, 20 g/kg of feed. Mean±SD, ^a-c^different letters on values in the same row indicate a significant difference (p<0.05), ^ns^values do not differ significantly (p≥0.05). CLE=Cashew leaf extract, SD=Standard deviation, VL=Villus length, CD=Crypt depth

## Discussion

The digestive system plays a significant role in livestock growth, and good nutrient absorption in chickens will result in high-quality poultry products. One of the most important organs for nutrient absorption is the small intestine, which can be used as a growth mechanism indicator [14]. A wide area of villi and a large number of goblet cells enhance nutrient absorption ability in livestock. The villi grow longer due to mitotic division in the small intestine epithelium, and their elongation widens the intestine area and consequently increases nutrient absorption [7].

The depth of intestinal crypts indicates the occurrence of cell division acceleration because when cell division occurs, the crypt cells will migrate and become epithelial cells. The depth of intestinal crypts also indicates the response to tissue exfoliation, inflammation, or toxin production by pathogens [7]. Goblet cells function to produce mucus, which plays an important role in brush border protection during food absorption [13,15].

Antimicrobials have been proven to inhibit one or more of the host responses to pathogens, such as inflammation, chemotaxis, reactive oxygen species production, and pro-inflammatory cytokine production [16,17]. Flavonoids have been reported to function as antioxidants, growth promoters, and ­antibacterial compounds [18]. Previous studies reported that ethanolic extracts of cashew leaf contain various secondary metabolites such as flavonoids, tannins, saponins, anthocyanins, and alkaloids [5]. Flavonoids in CLE may increase chicken appetite by promoting the release of pro-inflammatory cytokines [19]. Flavonoid, tannin, and saponin compounds in the ethanolic CLE can protect the walls of the gastric mucosa and small intestine. A properly protected gastric mucosal and small intestinal wall can improve nutrient absorption in animal feed [20].

Growth hormone (GH) stimulates protein synthesis and the absorption of amino acids that function in growth. The GH effect is directly mediated by insulin-like growth factor (IGF), which works on target cells to stimulate the growth of soft and bone tissues. The hormone IGF exerts its effect primarily by binding to an enzyme receptor that activates certain effector proteins within the target cell through phosphorylation of tyrosine residues [21]. Flavonoids may increase the weight of chickens by regulating GH and increasing IGF-I production. Flavonoids also trigger protein synthesis in muscles, which can enhance growth. The flavonoid content of ethanolic CLE may promote IGF-I production. GH through IGF-I stimulates the proliferation of epiphyseal cartilage, resulting in much space for bone formation, and stimulates osteoblasts in the growth process of chickens [22].

FCR in *Jawa Super* chickens given feed treated with CLE showed an improvement in the P1, P3, P4, and P5 group compared with the control and P2 groups. This indicates that CLE positively influenced growth performance and feed efficiency in *Jawa Super* chickens treated until 16 days of age. Our results suggest that a large villus area and CD in the duodenum will lead to a high activity of absorptive cells in absorbing feed nutrition. As a natural antibiotic, the content of ethanolic CLE can control and limit the growth and colonization of various types of pathogenic bacteria in a chick’s intestine. This feature can lead to higher efficiency in digestion and feed utilization, resulting in enhanced growth and improvement [23]. Cashew leaves inhibit the growth rate of Gram-positive and Gram-negative bacteria [24]. In one treatment group, Table-2 shows that the increase of P2 weight in the early phase was lower than that in the control group. This suggests that the P2 group needed to adapt to cashew extract in these early days. However, starting on day 9, weight gain in the P2 group increased more rapidly than in the control group.

Goblet cells in the duodenum are less numerous than those in the jejunum and ileum. In the duodenum, the number of goblet cells in P4 was significantly higher than in the controls. The area of goblet cells in the P4 duodenum was also significantly larger than that in the control. Goblet cells synthesize and secrete mucus consisting of glycoproteins. This synthesis of mucus is needed to raise the capacity of the mucus in the lumen so that pathogens fail to penetrate the tissue. When the secreted mucus is released into the lumen, the glycoprotein of the goblet cell mucus will form a useful gel as a protective barrier for epithelial cells. Goblet cells act as a regulator of epithelial hydration and interact with secretory IgA to produce antitoxin effects. Epithelial cells are protected from physical damage by intraluminal substances and block the invasion of pathogenic bacteria. To provide continuous protection, goblet cells must constantly be renewed [25,26].

Antimicrobial components such as flavonoids and tannins in ethanolic CLE are thought to prevent damage to goblet cells, so the number and size of mucus-producing goblet cells were higher in the treatment group than in the control. Tannins in cashew leaf can act as antimicrobial and fungicidal agents in chicken. Ethanolic CLE containing tannins can thus serve as a natural antimicrobial agent without reducing the availability of nutrients in the small intestine [27].

Goblet cells in the duodenum, jejunum, and ileum are dominated by cell types that produce acid mucin, which is colored blue through PAS–AB staining. Acid mucin is more effective in protecting the intestine from the translocation of pathogenic bacteria [15,28]. The results showed that the jejunum and ileum underwent similar morphological changes to the duodenum when treated with CLE. The absorption of food nutrition is higher in the duodenum than in the jejunum and ileum [1]. Absorptive cells are common in the duodenum region, whereas goblet cells are common in the ileum [29]. Quercetin and glycosides have also been used as antibacterial and antiviral agents [6]. These compounds are thought to influence the process of villus growth in the jejunum of the small intestine by protecting the villus area from damage due to attack from incoming pathogens.

## Conclusion

Administration of an ethanolic extract of cashew leaf at a dose of 10 g/kg of basal feed improves growth, body weight, and chicken feed efficiency of *Jawa Super* chickens. Treatment with the extract at 10 g/kg in the feed increased the length of villi, ratio of villi/crypts, number of goblet cells, and area of goblet cells in the small intestine of *Jawa Super* chickens compared with the untreated control.

## Authors’ Contributions

HS and MEJ contributed equally to the experimentation. HS and HTS wrote and edited the article. HTS and HS equally designed the experiment. All authors read and approved the final manuscript.
